# The Successful Management of a Repetitively Infected Recurrent Proximal Humerus Giant Cell Tumour of 20 Years' Duration With Two-Staged Surgery: A Rare Case Report

**DOI:** 10.7759/cureus.14492

**Published:** 2021-04-14

**Authors:** Alok C Agrawal, Ranjeet Choudhary, Shilp Verma

**Affiliations:** 1 Orthopaedics, All India Institute of Medical Sciences, Raipur, Raipur, IND; 2 Orthopaedics and Trauma, All India Institute of Medical Sciences, Raipur, Raipur, IND; 3 Orthopaedics and Trauma, All India Institute of Medical Sciences, Jodhpur, Jodhpur, IND; 4 Orthopaedic Surgery, All India Institute of Medical Sciences, Raipur, Raipur, IND

**Keywords:** giant cell tumour, recurrence, infection, proximal humerus, two-stage revision and reconstruction

## Abstract

The recurrence of giant cell tumour of bone (GCTB) is quite well known. It is mainly attributed to the presence of microscopic tumour remnants left behind after tumour treatment by intralesional curettage. This condition becomes more serious and alarming when the lesion gets infected postoperatively. Several studies have indicated that the role of adjuvants in preventing the recurrence of GCTs is limited, and complete removal of malignant cells is often mandatory. We present a rare case GCT of the proximal humerus in a female patient who developed repetitive recurrences of the tumour; her salvage procedures were also complicated by the development of infection after every treatment procedure for over 20 years. The patient was finally treated successfully with a two-stage revision and reconstruction procedure.

## Introduction

A giant cell tumour (GCT) is a benign aggressive bone lesion, which accounts for approximately 5% of all primary bone tumours; it has a female predominance. It is commonly observed between the third and fifth decades of life, but a small number of cases have been reported in individuals before the third decade (3%) or after the fifth decade (18%) [1,2]. It most commonly occurs in the epiphysio-metaphyseal region of long bones such as the distal femur, proximal tibia, and distal radius, and approximately 4% of the tumours are found in the proximal humerus region [3]. Patients usually present with primary complaints of pain, swelling, and functional disability [1-4]. Histopathologically, it is composed of the stroma of mononuclear spindle cells with dispersed multinucleated giant cells. Mononuclear cells are neoplastic and secrete substances such as osteoprotegerin ligand (OPGL), promoting the formation of benign multinucleated giant cells [4]. Although GCT is a benign condition, it can be locally aggressive with a high recurrence rate. Among all the reported cases, approximately 77% fall under Grade-II (well-defined margin but no radiopaque border) and 22% under Grade III (poorly defined border and impregnated in surrounding tissues) of the Enneking staging system [3]. Of note, 1% of apparently benign GCTs develop hematogenous metastases, most commonly associated with lungs [3].

The standard treatment for GCT is surgical resection of the tumour. The magnitude of resection depends on the tumour's location, size, grade, and biological aggression. Although curettage or intralesional excision is employed in most cases to control the disease while preserving maximal function, it often leaves behind microscopic foci of diseased tissue in the bone, which is responsible for a high recurrence rate. Campanacci et al. reported a local recurrence rate of 27% with intralesional procedures, 8% with marginal excisions, but no recurrence with wide local excisions [3]. Various curettage-associated adjuvant therapies are available to potentially reduce the risk of recurrences, such as phenol, polymethyl methacrylate, liquid nitrogen, and argon beam laser [5,6]. Some studies have questioned the role of adjuvants in reducing the recurrence rate of GCTs and stressed on the adequate removal of the tumour to improve the outcome of surgery [7,8].

In most cases, recurrence after curettage is diagnosed within two years of the primary intervention [3,9]. The treatment strategy for local recurrence is extensive curettage with cementation or wide resection of the lesion. Reconstruction of the bone void is performed with polymethylmethacrylate (PMMA), auto bone graft, or prosthesis based on the defect's location and extent. All the local adjuvant agents and filling materials are associated with their own disadvantages and complications. For instance, PMMA cementation is associated with immediate complications such as leaking cement into surrounding joints or soft tissues and delayed complications of osteoarthritic changes, slackness, and infection [9]. Several cases of GCT of the proximal humerus managed by extended curettage, bone cementation, and mega prosthetic reconstruction have been described in the literature. In this report, we discuss the management of a unique case of GCT of the proximal humerus that had repetitive recurrences and infections after every treatment procedure. The condition was finally managed with a two-step procedure. Since this entire course with multiple events happened over a period of 20 years, it provides an idea about the risk of recurrence and infection after the treatment of GCTs.

## Case presentation

**Figure 1:**
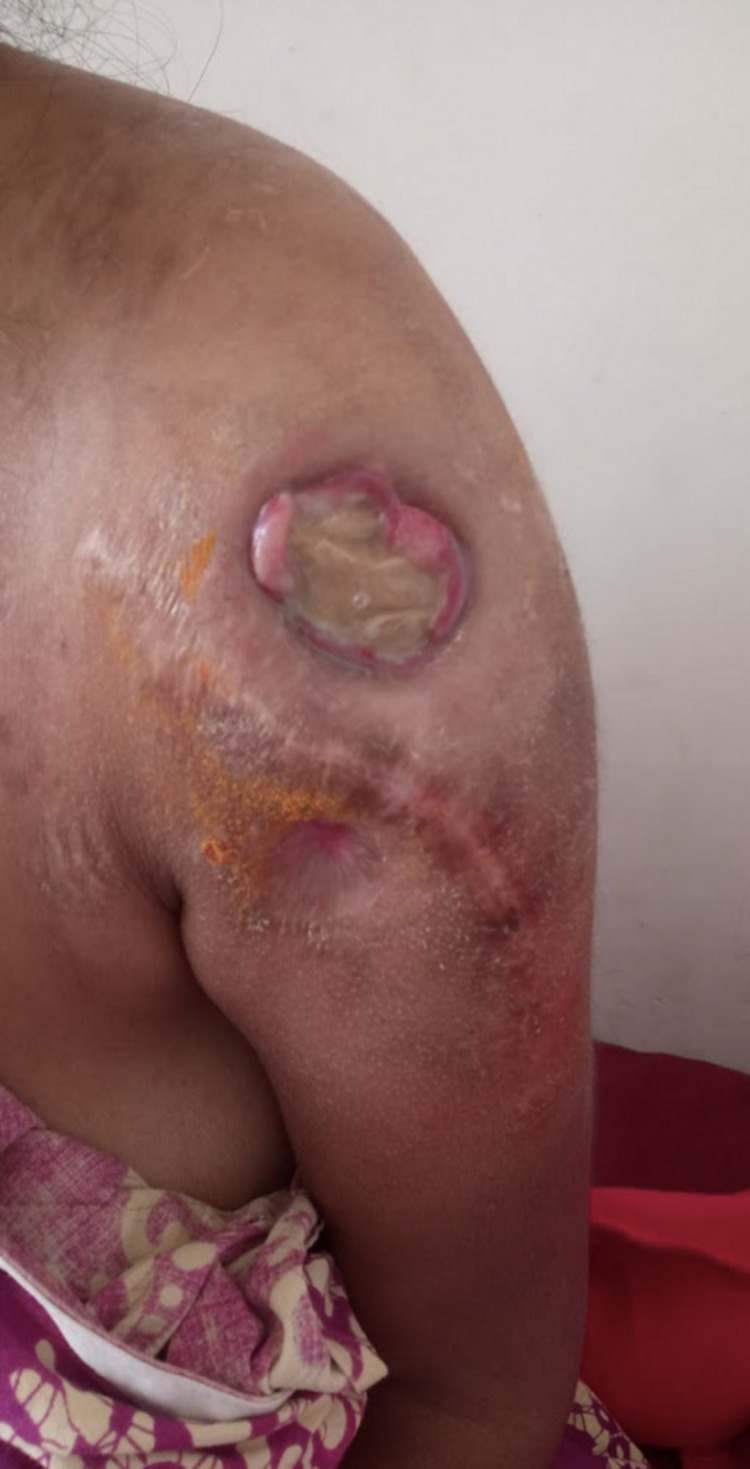
Clinical photograph of the patient presenting in our OPD with infected foul-smelling wound with exposed bone cement for several years (2018) OPD: outpatient department

A 40-year-old female patient presented to our Orthopedic department with complaints of pain, infection, and foul-smelling discharge from the left shoulder region along with limitations in left shoulder joint movements (Figure 1).

**Figure 2:**
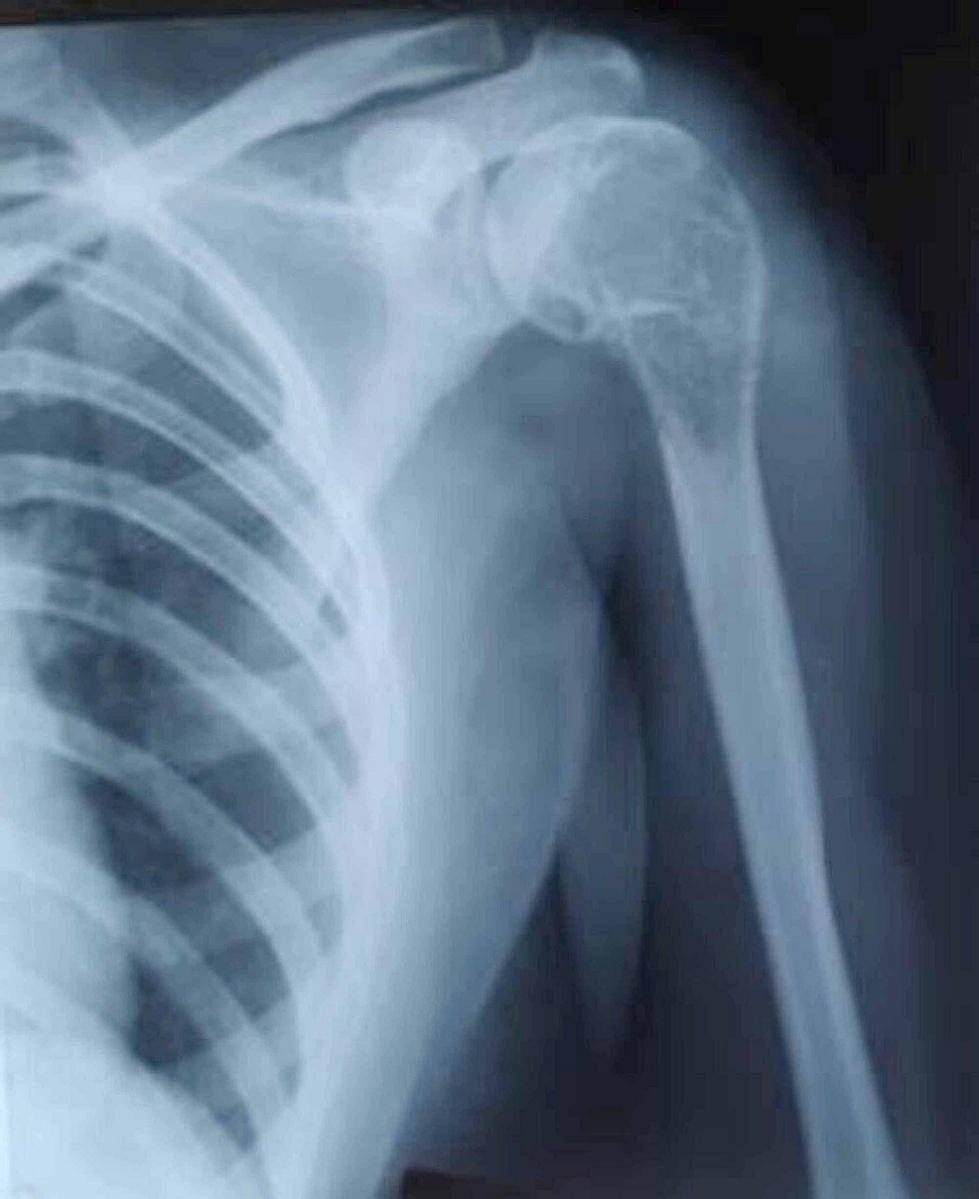
Preoperative X-ray of a giant cell tumour of the left proximal humerus around 20 years ago (2000)

She was facing social stigma and boycott due to her ailment. A detailed past medical history from the patient revealed a history of multiple surgeries in the left shoulder region from different hospitals. Around 20 years ago (2000), the patient had experienced pain in the left shoulder following trivial trauma. On radiological evaluation, it had been diagnosed as a pathological fracture with a well-defined lytic lesion in the proximal humerus (Figure 2).

**Figure 3:**
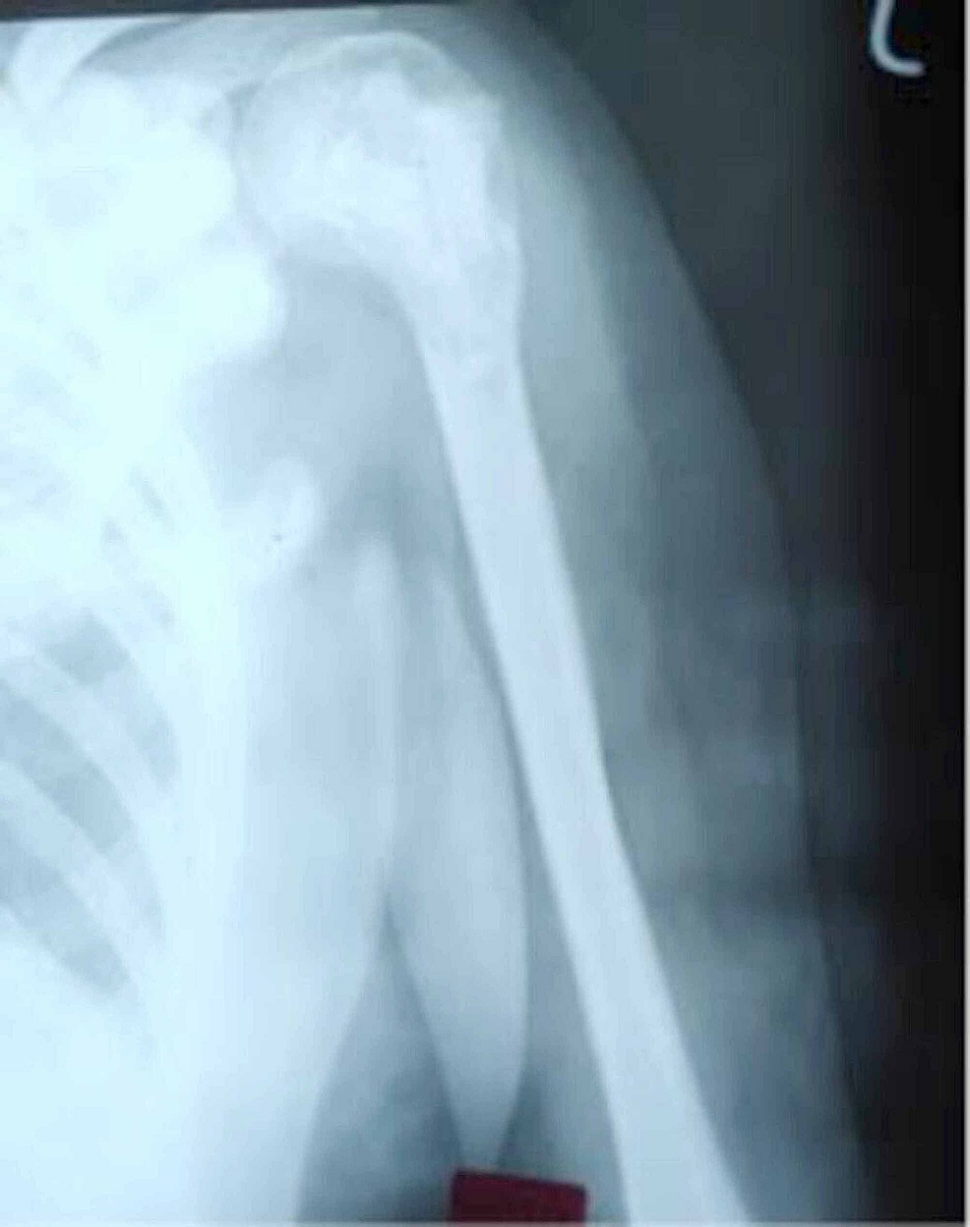
Postoperative X-ray following curettage and bone grafting (2001)

The lesion had been treated with curettage and iliac crest grafting at that time. The histological examination of curettage tissue had confirmed the diagnosis of GCT (Figure 3).

**Figure 4:**
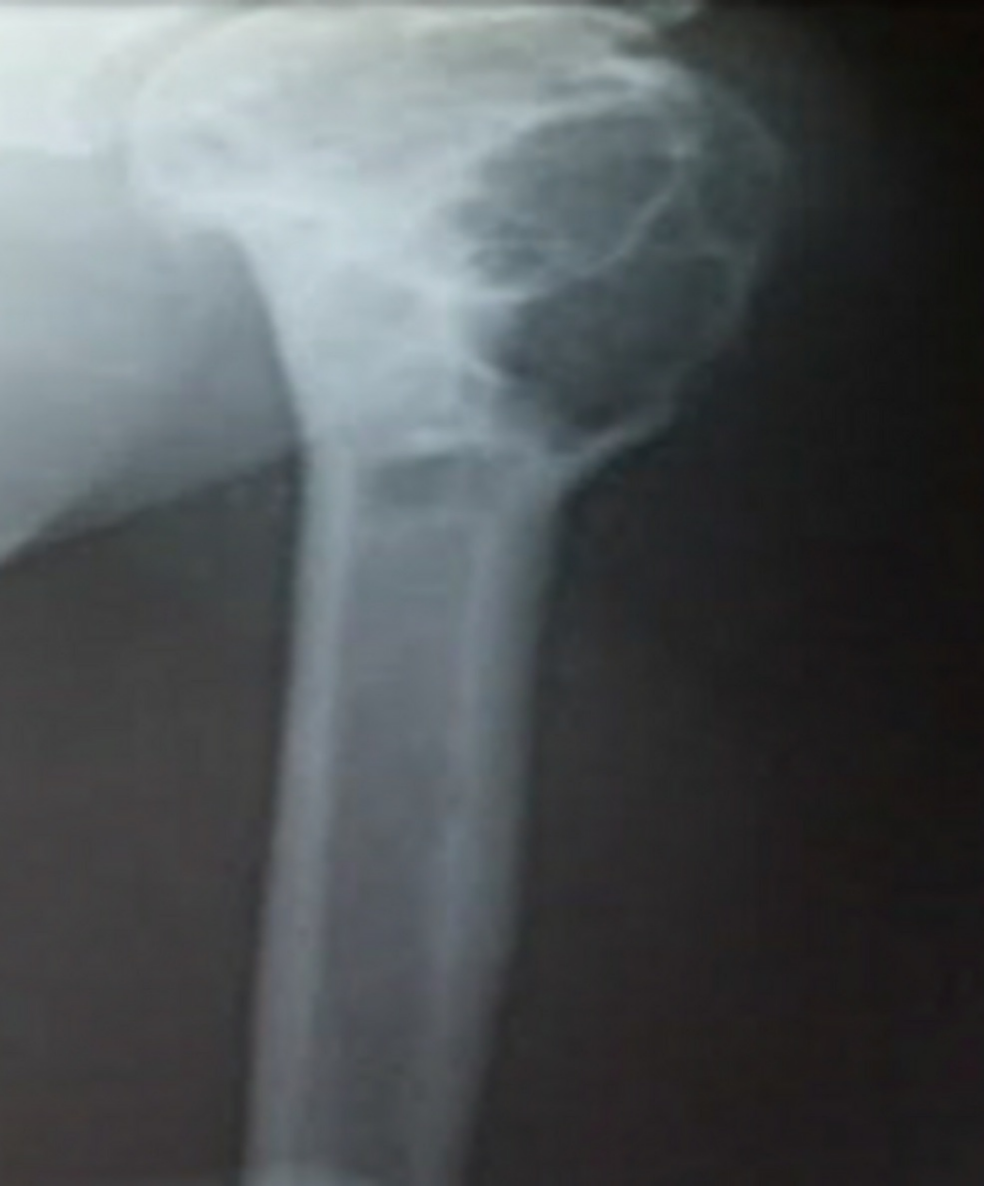
Preoperative X-ray showing recurrence for the first time following previous curettage and grafting (2003)

After treatment, the patient had been almost asymptomatic for three years when, in 2003, she had again developed gradual onset of pain in the same region, which had been diagnosed as a case of recurrent GCT on radiological assessment (Figure 4).

**Figure 5:**
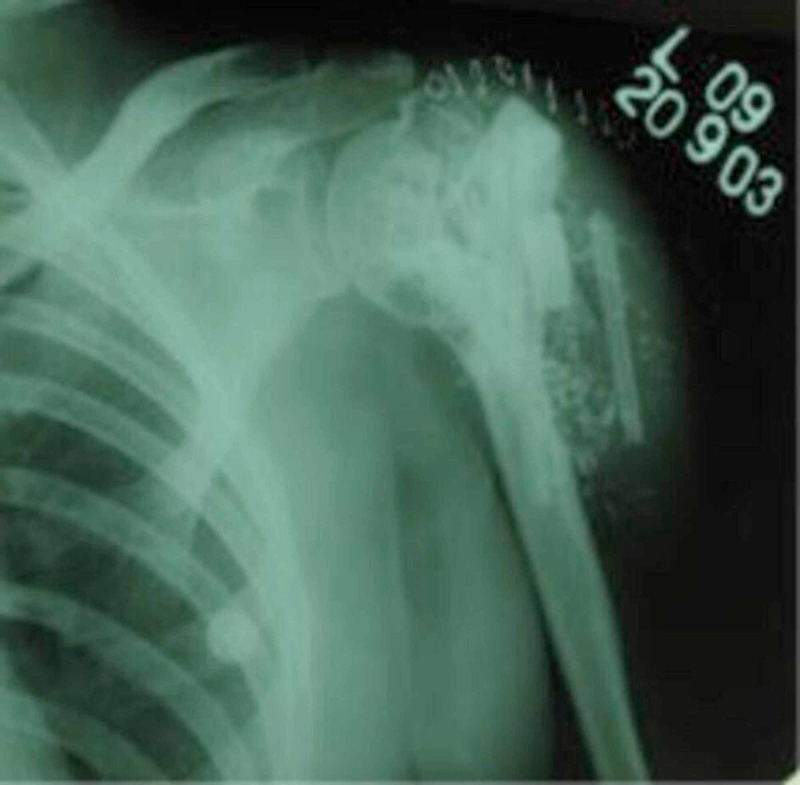
Postoperative X-ray showing repeat surgery done by curettage and fibular strut grafting (2003)

The patient had then undergone extended curettage followed by fibular strut graft and bone substitutes filling in the bone void (Figure 5).

**Figure 6:**
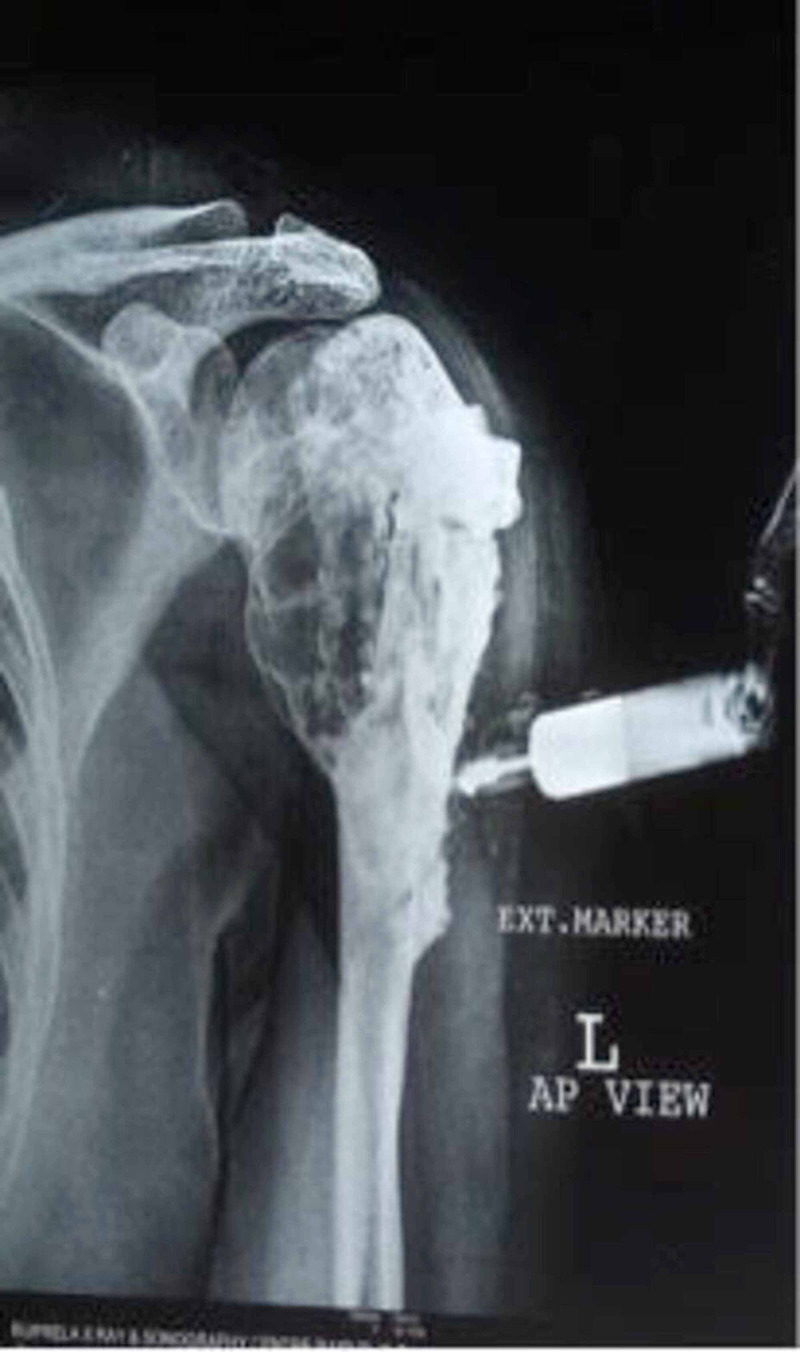
Preoperative sinogram taken in 2008 showing recurrence again with radioopaque dye spread throughout the lesion suggestive of frank infection

Postoperatively, the patient had developed an infection; she had been discharged and managed with conservative treatment (antibiotics and dressing). However, five years later (2008), the tumour had recurred again (Figure 6).

**Figure 7:**
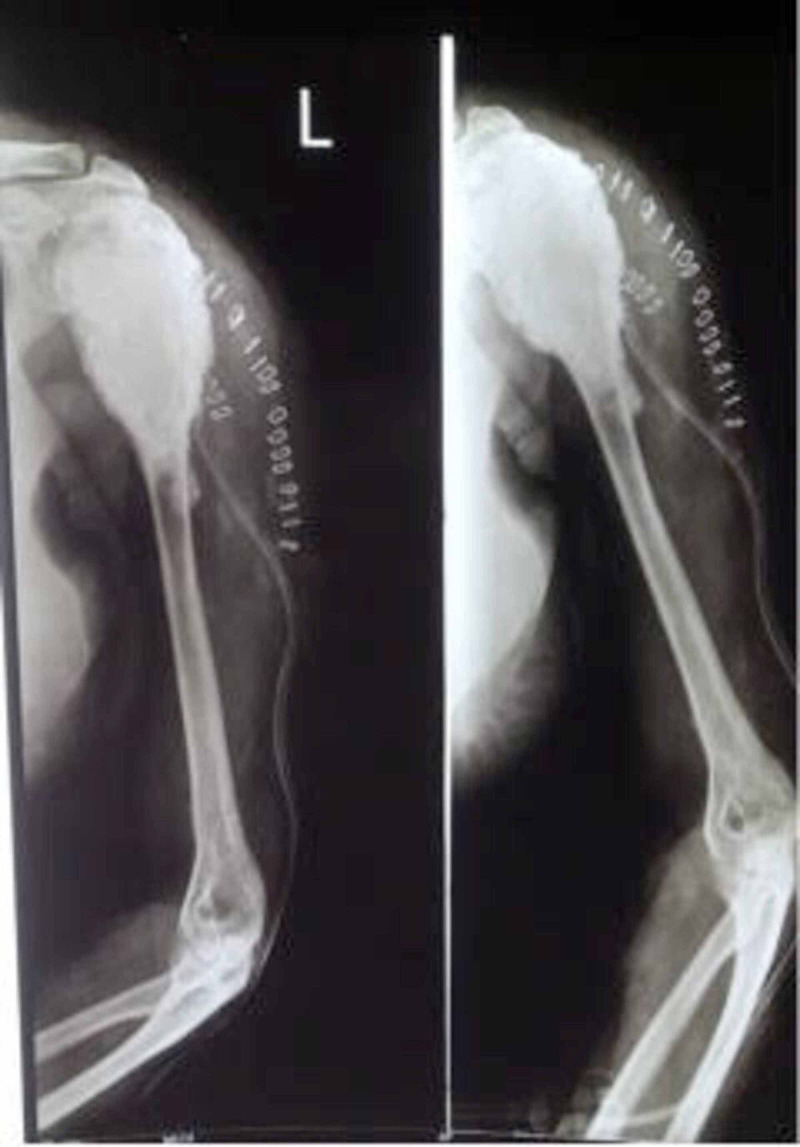
Postoperative X-ray showing the second recurrence managed by curettage and antibiotic cementation (2008)

Extensive curettage and cementation of the cavity were performed (Figure 7).

However, postoperative infection occurred this time as well, which progressed to a persistently draining sinus. Due to repetitive infections, the patient had lost hope and enthusiasm and had not approached any hospital for the next 10 years when she presented herself to our OPD in 2018 with a complaint of discharging wound and skin defect in the left shoulder region.

**Figure 8:**
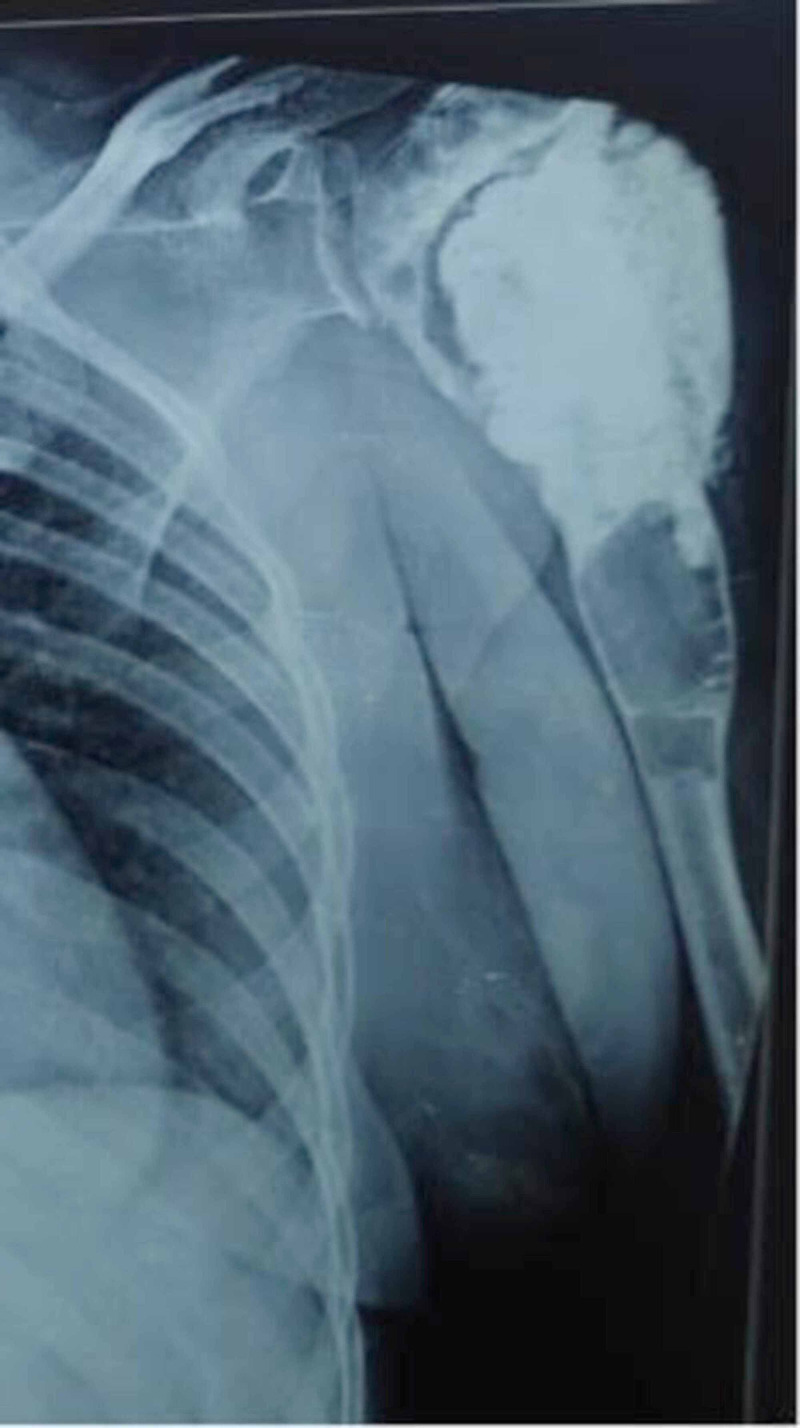
X-ray of the left shoulder showing bone cement separated by a radiolucent zone and osteolytic expansion of the diaphysis below suggestive of third recurrence (2018)

A non-healing wound of approximately 5 x 5 cm with underlying exposed bone cement and purulent discharge was present in the left deltoid region on clinical examination (Figure 1). Scar marks of previous surgeries were also present. Radiologic, hematologic, and microbiology investigations were performed. The radiograph showed a cavity filled with bone cement in the proximal humerus with a radiolucent area abutting its lower end (Figure 8).

Inflammatory markers such as erythrocyte sedimentation rate (ESR) and C-reactive protein (CRP) were also raised. Methicillin-sensitive *Staphylococcus aureus* (MSSA) and *Streptococcus pyogenes* grew in wound culture. The patient was started on intravenous clindamycin (300 mg qid) and cefuroxime (500 mg bid) for two weeks; however, the infection did not resolve. She was labeled as a case of infected recurrent GCT. A two-stage treatment was scheduled with a plan to eradicate the infection and excision of the lesion in the first stage, followed by the reconstruction of the defect with a customized mega prosthesis in the second stage.

First-stage operation

**Figure 9:**
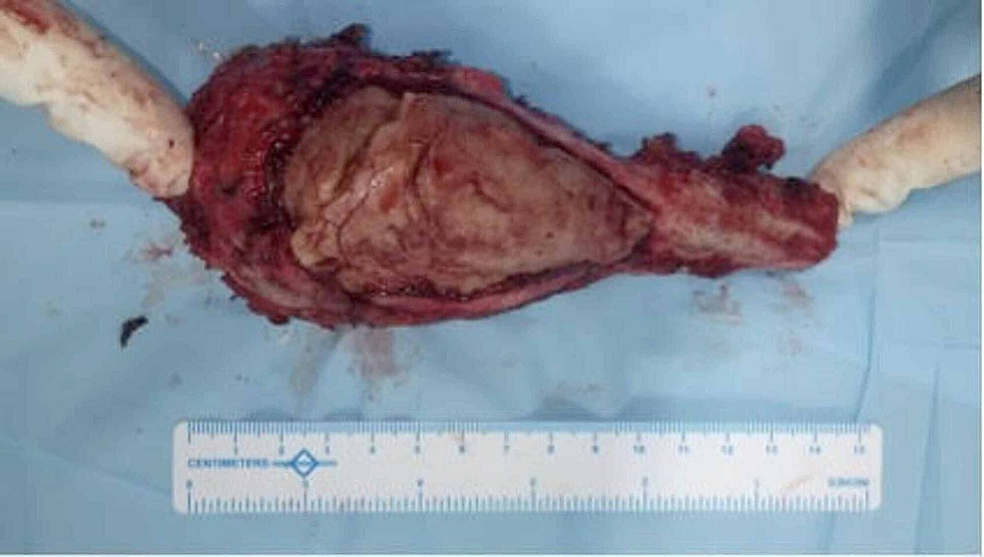
Intraoperative photograph showing excised proximal humerus and the bone cement within it measuring 6 inches in length

On the basis of preoperative radiological examination, wide local excision was planned including 1 cm of healthy bone margin. Under general anaesthesia, the patient underwent en-bloc excision (15 cms) of the proximal humerus and cement including a 5-mm healthy skin margin around the wound through the previously healed scar of the deltopectoral approach (Figure 9).

**Figure 10:**
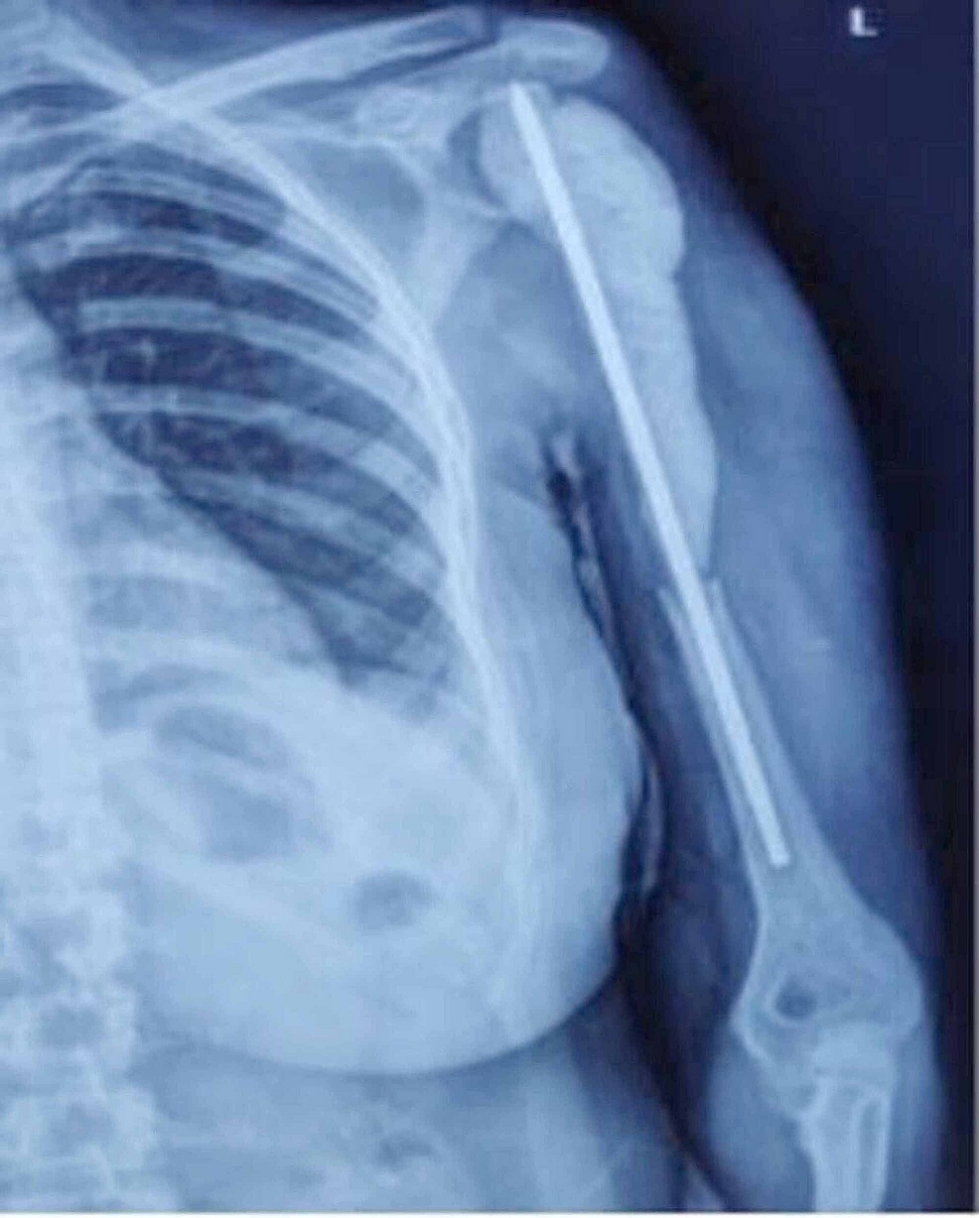
Postoperative X-ray showing void in bone filled with indigenously made antibiotic spacer over a Steinmann pin

Extensive debridement of nonviable infected tissue was done, including scared deltoid and rotator cuff. The wound (bone void) was thoroughly irrigated with 5% povidone-iodine and hydrogen peroxide solution and temporarily filled with an antibiotic spacer. The antibiotic spacer was made on the OT table with bone cement that contained vancomycin (4 g/40 mg), gentamicin (80 mg in 2-ml solution), and Steinmann pin (4.5) as the core (Figure 10).

Multiple Provisional holes were created in its proximal part for temporary fixation of muscle to maintaining the length of muscle and capsule cover. The wound was closed in a layered manner over a negative suction drain to prevent postoperative collection formation and removed after 48 hours. Antibiotic spacer eluted antibiotics in the local tissue environment, which helped to control the infection. Postoperatively, the patient did not report any local or systemic complications. There was no wound dehiscence or discharge, and the suture line had healed completely. Her ESR and CRP also reduced to baseline levels. The patient was instructed to report after three months for the second-stage surgery.

Second-stage operation

The proximal humerus region was exposed through the previous deltopectoral incision. An induced membrane was found around the spacer; however, there was no granulation tissue around it. Antibiotic cement spacer was removed, and the cavity was thoroughly cleaned with betadine and hydrogen peroxide aseptic solution. Distal humerus shaft was prepared with sequential reaming up to 7 mm in size. The bony defect was measured, and a modular proximal humerus reconstruction implant of appropriate size was selected (Restor humerus IM stem of 7-mm diameter and 80-mm length, Restor resection piece 75 mm, Restor humeral head; Modular Resection Prosthesis, Syncera, Smith & Nephew, London, UK). The implant's stem was fixed with PMMA gentamicin cement, and the rest of the implant components were assembled in appropriate rotation. The shoulder joint was reduced and covered with the fibrous capsule. Skin and subcutaneous tissues were closed in a layered manner over a drain. The drain was removed two days after surgery. No postoperative complications were present in the patient. Hand and elbow physiotherapy was started on the second postoperative day. However, the shoulder was kept immobilized with a brace for three weeks before physiotherapy.

Patient follow-up after 2.5 years (2020)

**Figure 11:**
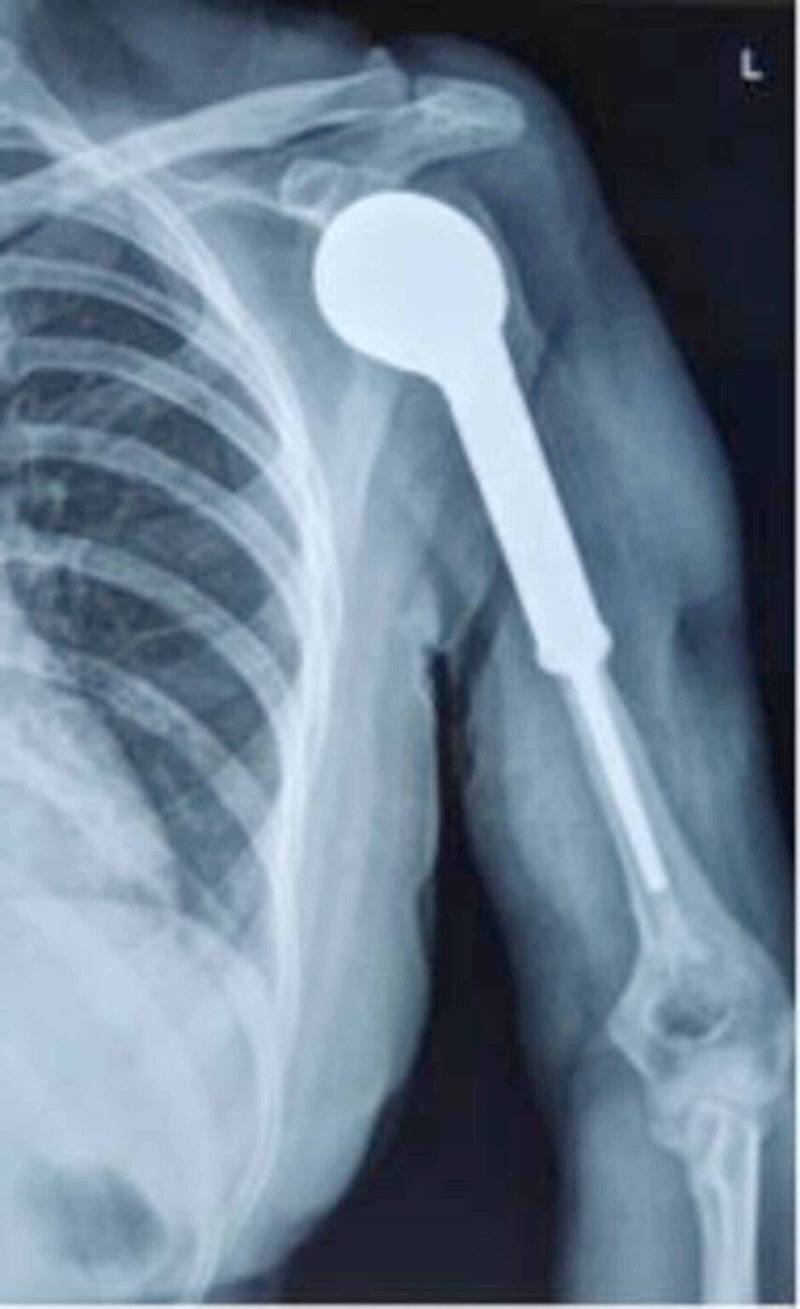
Postoperative follow-up X-ray in 2020 showing Restor Mega Prosthesis for the proximal humerus in position and with good fixation

After 2.5 years of treatment at our institution, the patient is currently asymptomatic, and the suture line is healthy. She does not have any clinical signs of infection and has a full range of motion at hand and elbow. The shoulder joint is stable; however, there is no active movement. The radiograph below shows the humerus stem well-seated without any cement-bone interface radiolucency or lysis of the bone (Figure 11).

**Figure 12:**
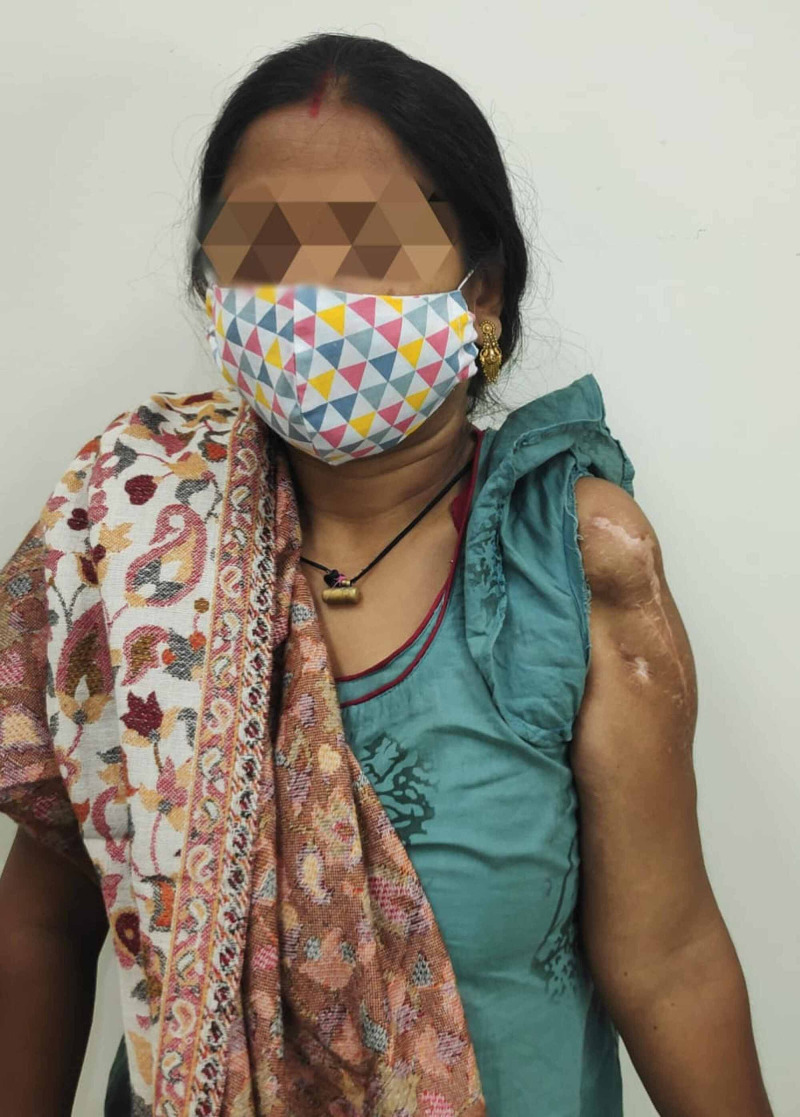
Clinical photograph of the patient’s stable shoulder

Inflammatory markers such as ESR and CRP are within the normal range. She is able to perform household activities and personal care without any assistance. Figure 12 shows the clinical photograph of the patient’s stable shoulder.

## Discussion

Recurrence of GCT is a well-known complication, and it mostly presents within two years of primary treatment. The incidence of local recurrence after treatment with curettage followed by either cementation or bone grafting is higher than that with en-bloc resection [3]. Campanacci et al. reported a local recurrence rate of 27% with intralesional procedures [3]. In the present case report, GCT in the proximal humerus had multiple episodes of recurrence and infection over a period of 20 years. Curettage and bone grafting were performed initially and after the first recurrence of the tumour. After the second recurrence, extended curettage and bone cementing were done.

However, unfortunately, the patient again developed an infection and experienced recurrence. A Scandinavian Sarcoma Group study has reported that 14% of patients with GCT had at least one local recurrence, and approximately two-thirds of patients with local recurrence were successfully treated with curettage and cementing [10-14]. This same study also mentioned a proximal humerus GCT local recurrence case treated with wide local excision and endoprosthesis [14].

Intralesional curettage is a standard treatment for GCT, using bone grafting/PMMA cement. Large and juxta-articular lesions are not suitable for bone grafting due to the unavailability of a large amount of bone graft. Bone grafting alone does not provide immediate subchondral strength, which might lead to chondral fracture or failure. Also, bone grafting is inherently associated with the risk of local infection. PMMA reconstruction of the defect after intralesional curettage restores subchondral stiffness within 98% of the contralateral limb [10]. Besides its use for the reconstruction of the defect, PMMA cement is also believed to act as an adjuvant due to its exothermic reaction and toxic effects on tumour cells. Some studies have shown a decreased rate of recurrence following PMMA cementation [7,8]. In the Scandinavian Sarcoma Group study, a lower local recurrence rate was reported with PMMA cementation than without cementation (20% vs. 56%, p=0.001) [11]. In contrast, in the Canadian Sarcoma Group study, the use of adjuvants for filling the cavity failed to show any statistical impact on the recurrence rate [12]. Therefore, it is assumed that adequacy of excision rather than the use of adjuvants is the determining factor in the recurrence of the tumour [8].

One disadvantage of using cement close to the articular surface is the risk of articular cartilage damage and early onset of joint degeneration. Bini et al. reported that 11% of patients experienced degenerative arthritis after subchondral cementing [15]. However, Wada et al. reported only one case with acrylic cement reconstruction where the patient developed osteoarthritis of the knee joint 14 years postoperatively. They emphasized that the continuity of articular cartilage at the time of surgery is more important [16]. Bone-cement interface radiolucent zones increased in width up to 2.5 mm during the first six months and were surrounded by a sclerotic rim, which is normal and probably caused by thermal burn injury of the surrounding bone [16].

In our case, en-bloc excision of the proximal humerus had led to the insufficiency of the rotator cuff and deltoid muscle. So we were unable to perform reverse shoulder arthroplasty due to the absence of functional deltoid muscle, which is essential for reverse shoulder arthroplasty. Hence, proximal humerus reconstruction with modular mega prosthesis was chosen as the method. Postoperatively, the patient has had a pain-free and emotionally acceptable limb with a full range of motion at hand and elbow; however, she is unable to actively perform movements at the shoulder. Another possible option was to achieve a stable shoulder joint arthrodesis, but considering the large proximal humerus defect, it could not be performed. Fuhrmann et al. have reported about the replacement of proximal humerus tumour with a modular endoprosthesis. They concluded that the modular endoprosthesis acts like a spacer and provides pain relief and emotional acceptance, preserves arm length, and allows the distal joints' unrestricted motion, but does not improve the active range of shoulder motion [17]. The functional outcome of our work is similar to that of Fuhrmann et al.'s study [17].

## Conclusions

GCT recurrence is believed to be due to microscopic residual tumour cells following intralesional curettage. While the likelihood of recurrence is minimized with the help of adjuvant treatment, recurrence has not been entirely eliminated. The first instance recurrence is normally treated with intralesional curettage and cementation, but wide local excision is required for treating repeat recurrence. It is impossible to control as recurrence is complicated by deep infection. We presented an unusual case of proximal humerus giant cell tumour, which had multiple infection recurrences over a 20-year period. The two-stage management we discussed in this report could be effective in handling this form of GCT recurrence and ensuring limb protection.
